# D‐Dimer: A Mediator of the Association Between Lymphocyte and Dissemination of Pulmonary Tuberculosis: A Retrospective Cohort Study

**DOI:** 10.1111/crj.70175

**Published:** 2026-03-17

**Authors:** Yujun Lin, Di Wu, Xiaohong Chen, Lujing Jiang, Yiming Zeng

**Affiliations:** ^1^ Department of Pulmonary and Critical Care Medicine, The Second Affiliated Hospital Fujian Medical University Quanzhou City Fujian China; ^2^ Department of Pulmonary and Critical Care Medicine Fujian Medical University Clinical Teaching Hospital, Fuzhou Pulmonary Hospital of Fujian Province Fuzhou City Fujian China; ^3^ Central Laboratory Fujian Medical University Clinical Teaching Hospital, Fuzhou Pulmonary Hospital of Fujian Province Fuzhou City Fujian China; ^4^ Department of Tuberculosis Fujian Medical University Clinical Teaching Hospital, Fuzhou Pulmonary Hospital of Fujian Province Fuzhou City Fujian China; ^5^ Department of Pharmacy Fujian Medical University Clinical Teaching Hospital, Fuzhou Pulmonary Hospital of Fujian Province Fuzhou Fujian Province China; ^6^ Fujian Key Laboratory of Lung Stem Cell, the Second Affiliated Hospital Fujian Medical University Quanzhou City Fujian China

**Keywords:** cross‐sectional study, D‐dimer, extrapulmonary tuberculosis, mediation analysis, pulmonary tuberculosis

## Abstract

**Introduction:**

This study aimed to examine whether D‐dimer and lymphocyte counts predict the risk of concurrent pulmonary tuberculosis (PTB) and extrapulmonary tuberculosis (EPTB) and to identify critical thresholds for clinical use. We also investigated whether D‐dimer mediates the protective effect of lymphocytes against tuberculosis (TB) dissemination.

**Methods:**

One thousand nine hundred (1318 PTB and 582 PTB + EPTB) patients diagnosed between 2022 and 2024 were analyzed. Multiple regression analysis, smooth curve fitting, threshold effect analysis, and causal mediation analysis were conducted using EasyStat and R software to evaluate the association between lymphocyte counts (exposure), D‐dimer (mediator), and PTB + EPTB risk (outcome) and to determine the critical value of lymphocyte counts and D‐dimer.

**Results:**

PTB + EPTB patients had higher D‐dimer and lower lymphocyte counts. Elevated D‐dimer increased the risk of PTB + EPTB (adjusted OR = 2.28, 95% CI: 1.99–2.60). High lymphocyte counts reduced the risk (adjusted OR = 0.25, 95% CI: 0.13–0.46). Threshold effects showed increased risk when D‐dimer exceeded 0.170 mg/L (OR = 2.35, 95% CI: 2.05–2.70) and reduced risk when lymphocyte counts exceeded 750 cells/μL (OR = 0.15, 95% CI: 0.07–0.32). D‐dimer mediated 36.473% (95% CI: 25.469–53.168) of the protective effect of lymphocytes.

**Conclusions:**

D‐dimer is an independent risk factor and lymphocyte counts a protective factor for PTB + EPTB, with D‐dimer mediating 36.473% of the lymphocyte effect. Clinically actionable thresholds (D‐dimer > 0.170 mg/L and lymphocytes < 750 cells/μL) provide concrete targets for early intervention to prevent TB dissemination and improve outcomes.

## Graphical abstract

This study identifies D‐dimer as a key mediator between lymphocyte depletion and pulmonary tuberculosis dissemination. Our findings reveal that reduced lymphocyte counts increase dissemination risk partly by elevating D‐dimer levels, suggesting a critical link between immune deficiency and coagulopathy. Monitoring D‐dimer may help clinicians predict and manage tuberculosis progression more effectively.

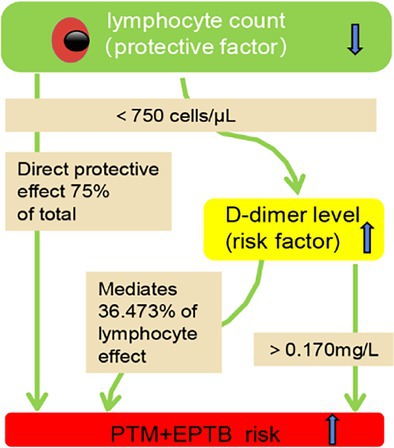

**Abbreviations**
ADA: adenosine deaminase
AFB: acid‐fast bacillus
ALB: albumin
BM: bacterial meningitis
CHOL: cholesterol
CI: confidence interval
CNS: central nervous system
COPD: chronic obstructive pulmonary disease
EPTB: extrapulmonary tuberculosis
FBG: fasting blood glucose
HGB: hemoglobin
LYM: lymphocyte count
M. tb:
*Mycobacterium tuberculosis*

NK cells: natural killer cells
NTM: nontuberculous mycobacteria
OR: odds ratio
PE: pulmonary embolism
PLT: platelet count
PTB: pulmonary tuberculosis
TB: tuberculosis
TM: tuberculous meningitis
T‐SPOT.TB T‐SPOT: tuberculosis test
VTE: venous thromboembolism
WBC: white blood cell count

## 1 Introduction

Tuberculosis has likely re‐emerged as the leading global cause of death from a single infectious agent in 2023 [1]. Extrapulmonary tuberculosis (EPTB) accounts for 15%–32.75% of all reported tuberculosis (TB) instances [2, 3, 4, 5], posing greater management challenges than pulmonary tuberculosis (PTB) due to its complex clinical presentation and treatment requirements. Key challenges include difficult diagnosis due to nonspecific symptoms [6], involvement of diverse anatomical sites [7], limited drug penetration into extrapulmonary tissues [8], higher rates of treatment failure and relapse [9], and increased prevalence among immunocompromised individuals, such as those with HIV, which exacerbates disease severity [10].

Lymphocyte subsets dynamically orchestrate the control of tuberculosis dissemination through specialized immune functions. The proportion of CD4^+^ T cells and the ratio of CD4^+^/CD8^+^ T cells decreased in TB patients with disseminated infection [11]. Single‐cell profiling identifies granzyme K‐expressing CD8^+^ T‐cell subsets that might contribute to protection against tuberculosis dissemination [12]. The immune landscape in severe patients was characterized by widespread immune exhaustion in Th1 and CD8^+^ T cells [13]. Antigen‐specific B cells direct T follicular‐like helper cells into lymphoid follicles to mediate *M. tuberculosis* control [14].

D‐dimer, a protein fragment from fibrin degradation, is traditionally used to assess thrombotic conditions but has also been linked to poor outcomes in infections and inflammation [15, 16, 17, 18]. In TB, elevated D‐dimer levels have been associated with thromboembolic risks, inflammation, and complications. Studies by Zhang [19], Mujaddad [20] and Wang [21] have shown that higher D‐dimer levels in TB patients correlate with increased thromboembolic risk, elevated inflammatory markers, and higher pulmonary artery systolic pressure. These findings suggest that D‐dimer may play a role in TB progression and complications.

Current evidence suggests lymphocyte depletion facilitates mycobacterial dissemination, while D‐dimer not only indicates thromboembolic risk but also reflects inflammatory status and complications in TB. However, consensus is lacking regarding whether D‐dimer is associated with TB dissemination and coagulopathy initiates or results from TB dissemination. Crucially, no studies have quantitatively examined D‐dimer's potential mediation between lymphopenia and dissemination, creating a fundamental knowledge gap in disseminated TB pathogenesis.

To address this gap, we conducted a retrospective cohort study of 1900 tuberculosis patients (2022–2024). We independently analyzed lymphocyte dynamics and D‐dimer levels as exposures, with incident combined pulmonary–extrapulmonary TB (PTB + EPTB) diagnosis during follow‐up as the primary outcome. Employing causal mediation analysis—a methodological advance beyond prior correlative studies—we quantified D‐dimer's intermediary role in the pathway between lymphopenia and TB dissemination. This approach identified critical coagulation biomarker thresholds directly informing clinical risk stratification, potentially enabling early interventions targeting fibrinolytic pathways to prevent dissemination in lymphopenia patients.

## 2 Methods

This retrospective cohort study enrolled 6481‐TB patients diagnosed between 1 January 2022 and 16 December 2024 at Fuzhou Pulmonary Hospital. The diagnosis of tuberculosis (TB) adhered to both the WHO guidelines [22] and the clinical diagnostic standards for TB established by the Chinese Medical Association [23]. In clinical practice, a combination of traditional and modern diagnostic methods was adopted, with comprehensive consideration of clinical manifestations, physical examination findings, and bacteriological test results to confirm TB. Patients were categorized into PTB (*n* = 4618) and PTB + EPTB (*n* = 1863), with the latter including complications like tuberculous pleuritis, peritonitis, meningitis, and other extrapulmonary manifestations. Exclusions included HIV, viral hepatitis, endocrine and metabolic diseases, rheumatic diseases, and other lung conditions such as chronic obstructive pulmonary disease (COPD) and asthma, pregnancy, nontuberculous mycobacteria (NTM) infections, tumors, coronary stent placement, pulmonary embolism, sepsis, and bilateral lower extremity venous thrombosis. After exclusions and removing cases with missing D‐dimer and lymphocyte subsets data, 1900 patients (1318 PTB and 582 PTB + EPTB) were analyzed (Figure S1). Clinical data included age, D‐dimer, complete blood counts, fasting blood glucose (FBG), albumin (ALB), cholesterol (CHOL), adenosine deaminase (ADA), AFB smear, T‐SPOT.TB test, and lymphocyte subsets (CD3^+^ T cells, CD4^+^ T cells, CD8^+^ T cells, and CD19^+^ B cells). The nonnormal distributions of multiple parameters were addressed through the application of natural logarithmic transformations to normalize their distributions. Statistical analyses included descriptive statistics, group comparisons, multivariable logistic regression, smooth curve fitting, and causal mediation analysis were performed by EasyStat software (X&Y solutions Inc. Version 5.0, Boston, MA, USA) and R software (The R Foundation for Statistical Computing, version 4.3.2, Vienna, Austria).

## 3 Results

### 3.1 Analysis of Clinical Characteristics of TB Patients

This study included 1900‐TB patients, with 1318 in the PTB group and 582 in the PTB + EPTB group. As shown in Table 1, the two groups had comparable age distributions: the mean (SD) age was 52.03 (17.67) years in the PTB group and 51.34 (19.88) years in the PTB + EPTB group (*p* = 0.453). The PTB + EPTB group had higher levels of ln D‐dimer (0.52 vs. −0.53, *p* < 0.001) and ADA (16.67 U/L vs. 14.47 U/L, *p* < 0.001). Conversely, the PTB + EPTB group had lower levels of ln lymphocytes count (0.02 vs. 0.13, *p* < 0.001), hemoglobin (116.64 g/L vs. 122.21 g/L, *p* < 0.001), serum albumin (36.41 g/L vs. 38.64 g/L, *p* < 0.001), and cholesterol (3.98 mmol/L vs. 4.28 mmol/L, *p* < 0.001). Immune cell profiles showed reduced levels of CD3^+^, CD4^+^, CD8^+^ T cells, CD19^+^ B cells, and NK cells in the PTB + EPTB group (all *p* < 0.001). Additionally, the PTB + EPTB group had a lower AFB smear positivity (19.93% vs. 31.56%, *p* < 0.001).

**TABLE 1:** Comparison of clinical and laboratory characteristics between PTB and PTB + EPTB groups.

Characteristic	PTB (*n* = 1318)	PTB + EPTB (*n* = 582)	*p*
D‐dimer (ln)	−0.53 (1.01) −0.60 (−4.61–3.63)	0.52 (1.18) 0.49 (−3.51–3.22)	< 0.001^**†**^
Age (years)	52.03 (17.67) 55.00 (0.90–91.00)	51.34 (19.88) 54.00 (0.00–97.00)	0.453^**†**^
WBC count (10^9/L)	7.25 (3.13) 6.75 (1.60–60.37)	6.95 (2.74) 6.38 (1.18–23.37)	0.046^**†**^
Lymphocyte count (ln)	0.13 (0.22) 0.16 (−1.15–0.91)	0.02 (0.23) 0.03 (−0.82–0.58)	< 0.001^**†**^
HGB (g/L)	122.21 (19.18) 123.00 (46.00–173.00)	116.64 (19.30) 117.00 (60.00–170.00)	< 0.001^**†**^
PLT (10^9/L)	277.33 (98.26) 260.00 (29.00–804.00)	315.99 (122.60) 302.00 (20.00–782.00)	< 0.001^**†**^
FBG (mmol/L)	6.46 (3.20) 5.20 (2.80–30.10)	6.19 (2.75) 5.26 (3.00–29.05)	0.806^**†**^
ALB (g/L)	38.65 (4.94) 39.20 (20.80–51.70)	36.41 (4.63) 36.70 (22.10–49.80)	< 0.001^**†**^
CHOL (mmol/L)	4.28 (1.01) 4.18 (1.14–10.06)	3.98 (0.90) 3.94 (1.27–7.04)	< 0.001^**†**^
ADA (U/L)	14.47 (7.38) 12.41 (3.90–62.04)	16.67 (7.22) 15.21 (5.57–55.00)	< 0.001^**†**^
CD3◻ T cells (cells/μL)	1172.91 (540.82) 1113.50 (82.00–3462.00)	880.64 (449.82) 799.50 (76.00–2744.00)	< 0.001^**†**^
CD4◻ T cells (ln)	6.39 (0.59) 6.48 (0.69–7.80)	6.06 (0.67) 6.15 (2.89–7.70)	< 0.001^**†**^
CD8◻ T cells (ln)	5.94 (0.59) 5.98 (3.37–7.45)	5.65 (0.61) 5.73 (3.14–7.35)	< 0.001^**†**^
CD19◻ T cells (ln)	5.19 (0.82) 5.30 (0.00–8.10)	4.85 (0.90) 4.96 (1.10–7.56)	< 0.001^**†**^
NK cells (ln)	5.18 (0.74) 5.18 (1.95–7.22)	4.92 (0.75) 4.94 (2.40–7.14)	< 0.001^**†**^
T‐SPOT.TB			0.753^**‡**^
Negative	143 (10.85%)	66 (11.34%)	
Positive	1175 (89.15%)	516 (88.66%)	
AFB smear (positive)			< 0.001**‡**
Negative	902(68.44%)	466 (80.07%)	
Positive	416 (31.56%)	116 (19.93%)	

*Note:* To address the nonnormal distribution of parameters (D‐dimer, lymphocyte, CD4^+^, CD8^+^, CD19^+^, and NK cells), natural logarithmic transformation was applied to normalize the distribution. Continuous variables are presented as mean (SD), median (Min–Max); categorical variables are presented as *N* (%). Data were analyzed by the ^†^Kruskal–Wallis rank sum test or ^‡^Fisher's exact test.

Abbreviations: ADA, adenosine deaminase; ALB, albumin; CHOL, cholesterol; FBG, fasting blood glucose; HGB, hemoglobin; NK cells, natural killer cells; PLT, platelet; WBC, white blood counts.

Overall, PTB + EPTB patients exhibited distinct clinical and immunological profiles, characterized by reduced immune cell counts, lower hemoglobin, albumin, and cholesterol levels and higher D‐dimer and ADA levels, suggesting a more severe systemic impact and altered immune response.

### 3.2 Multiple Regression Analysis Revealed That D‐Dimer and Lymphocyte Count Were Independently Associated With PTB Complicated by EPTB in a Linear Relationship

Multiple regression analysis (Table 2) demonstrated that higher ln D‐dimer levels are independently associated with an increased risk of PTB + EPTB (Model I). In the nonadjusted model, each unit increase in ln D‐dimer was linked to a 140% higher risk of adverse outcomes (OR: 2.40, 95% CI: 2.16–2.67, *p* < 0.001). This association remained significant after adjusting for covariates including Age, AFB smear, T‐SPOT.TB, WBC count, lymphocyte count, HGB, PLT count, FBG, ALB, CHOL, ADA, CD3^+^ T cells, CD4^+^ T cell, CD8^+^ T cell, CD19^+^ T cell, and natural killer cells (OR: 2.28, 95% CI: 1.99–2.60, *p* < 0.001).

**TABLE 2:** Associations of D‐dimer and lymphocyte counts levels with combined pulmonary and extrapulmonary tuberculosis.

Model	Exposure	Outcome	Nonadjusted	Adjust
I	ln D‐dimer	PTB + EPTB	2.40 (2.16, 2.67) < 0.001	2.28 (1.99, 2.60) < 0.001
II	ln lymphocyte count	PTB + EPTB	0.10(0.06, 0.15) < 0.001	0.25 (0.13,0.46) < 0.001

*Note:* Results are presented as odds ratios (OR) with 95% confidence intervals (CI) and *p*‐values. Variables were natural logarithmic transformations of D‐dimer and lymphocyte counts level.Adjust for in Model I: Age, AFB smear, T‐SPOT.TB, WBC, lymphocyte, HGB, PLT, FBG, ALB, CHOL, ADA, CD3^+^ T cell, CD4^+^ T cell, CD8^+^ T cell, CD19^+^ T cell, and NK cells. Adjust For in Model II: Age, AFB smear, T‐SPOT.TB, WBC, HGB, PLT, FBG, ALB, CHOL, D‐Dimer, ADA, and NK cells.

Abbreviations: ADA, adenosine deaminase; ALB: albumin; CHOL, cholesterol; FBG, fasting blood glucose; HGB, hemoglobin; NK cells, natural killer cells; PLT, platelet; WBC, white blood counts.

Sample sizes:

**Table:** 

Model	Non‐adjusted	Adjust
I	1900	1900
II	1900	1900

In Model II: Analysis demonstrated that lymphocyte counts were independently associated with PTB + EPTB outcomes in a protective relationship. Higher ln lymphocyte count levels were consistently associated with reduced risk of PTB + EPTB across all adjustment models. In the nonadjusted model, each unit increase in ln lymphocyte count corresponded to a 90% lower risk of PTB + EPTB (OR: 0.10, 95% CI: 0.06–0.15, *p* < 0.001). This protective association persisted after adjusting for covariates including AFB smear, T‐SPOT.TB, WBC count, HGB, PLT count, FBG, ALB, CHOL, D‐dimer, and NK cells (OR: 0.25, 95% CI: 0.13–0.46, *p* < 0.001).

### 3.3 Curve Fitting Analyses Reveal Distinct Associations Between ln D‐Dimer, Lymphocyte Counts, and PTB complicated by EPTB

The smooth curve fitting analysis (Figure S2A) shows a significant upward trend between ln D‐dimer levels and the risk of PTB complicated by EPTB, indicating its potential as a critical biomarker. The confidence intervals represented by the blue dotted lines provide a measure of uncertainty around the estimated risk, highlighting the variability in the data. The results suggest that monitoring D‐dimer levels could be essential for assessing the severity of disease and the likelihood of PTB complicated with EPTB, aligning with Table 2 Model I regression results.

The smooth curve fitting analysis for lymphocyte counts (Figure S2B) reveals a pronounced downward trend, indicating that higher basal ln lymphocyte counts are associated with a reduced risk of PTB complicated by EPTB. The red trend line shows a consistent decline in risk as ln lymphocyte counts increase. This visualization strongly supports lymphocyte count's role as a protective factor against PTB with EPTB, aligning with Table 2 Model II regression results.

To sum up, D‐dimer is a significant risk biomarker for PTB with EPTB, showing consistent positive associations across adjustment models. Conversely, lymphocyte count is a robust protective factor, with both biomarkers demonstrating clinically relevant monotonic relationships through curve fitting analyses.

### 3.4 Associations of ln D‐Dimer Levels and Lymphocyte Counts With Risk of PTB Complicated by EPTB Across Threshold and Linear Models

This analysis examined the relationship between exposure variables (ln D‐dimer, ln lymphocyte counts) and the outcome variable “PTB + EPTB” (Table 3). Model I^a^ revealed an odds ratio of 2.27 (95% CI: 1.99, 2.60; *p* < 0.001) for ln D‐dimer, indicating that each unit increase in ln D‐dimer corresponds to a 127% increase in the odds of being classified in the higher risk group. This strong association highlights D‐dimer as a significant biomarker for identifying patients at risk for complications.

**TABLE 3:** Associations of ln D‐Dimer and lymphocyte counts with tuberculosis dissemination risk: Linear and threshold regression models.

Model	Exposure variable	Outcome variable	Effect value (*β*)	95% Confidence interval (CI)	*p*
Model I^a^	ln D‐dimer	PTB + EPTB	2.27	(1.99, 2.60)	< 0.001
Model I^b^	ln lymphocyte count	PTB + EPTB	0.25	(0.13, 0.46)	< 0.001
Model II^a^	ln D‐dimer	PTB + EPTB	−1.77 (break point)	—	—
	< K		0.77	(0.33, 1.76)	0.531
	> K		2.35	(2.05, 2.70)	< 0.001
Model II^b^	ln lymphocyte count	PTB + EPTB	−0.29 (break point)	—	—
	< K		2.31	(0.33, 15.95)	0.397
	> K		0.15	(0.07, 0.32)	< 0.001

*Note:* K: Represents the break point identified in the analysis. The *p*‐value less than 0.05 considered significant.

Model II^a^ identified a break point (K) of ln D‐dimer at −1.77, equating to a D‐dimer value of approximately 0.170 mg/L. For D‐dimer levels below this threshold (< K), the odds ratio was 0.77 (95% CI: 0.33, 1.76; *p* = 0.531), suggesting no significant risk increase. In contrast, for levels above the break point (> K), the odds ratio rose to 2.35 (95% CI: 2.05, 2.70; *p* < 0.001), indicating a 135% increase in odds of higher classification.

The likelihood ratio test yielded a *p*‐value of 0.03, demonstrating that the model with the break point significantly improves fit compared to one without it. This underscores the importance of considering both the magnitude and context of D‐dimer levels in clinical assessments.

The linear Model I^b^ analysis showed an inverse relationship between lymphocyte counts and TB dissemination risk. Each unit increase in the ln lymphocyte counts was significantly associated with a 75% reduction in the odds of PTB complicated by EPTB (OR: 0.25; 95% CI: 0.13–0.46, *p* < 0.001). This indicates lymphocyte counts serve as a protective biomarker against disease dissemination.

The threshold regression analysis Model II^b^ identified a significant break point (K) at ln lymphocyte counts = −0.29, equating to a lymphocyte counts value of approximately 750 cells/μL. Below this threshold (< K), no significant association was observed (OR: 2.31; 95% CI: 0.33–15.95, *p* = 0.397). Above the break point (≥ K), lymphocyte counts exhibited strong protective effects: each unit increase reduced odds of PTB + EPTB by 85% (OR: 0.15; 95% CI: 0.07–0.32, *p* < 0.001).

The likelihood ratio test *p*‐value of 0.016 provides strong evidence (at the 5% significance level) that the threshold model with a break point fits the data significantly better than a simple linear model. This confirms that lymphocyte counts exhibit a nonlinear relationship with tuberculosis dissemination risk, where the effect changes substantially below versus above the critical threshold (K = −0.29 on the ln scale).

### 3.5 Multiple Regression Analysis Revealed That Lymphocyte Count Was Independently Associated With D‐Dimer

To examine the association between lymphocyte count and D‐dimer levels, we implemented a threshold‐based categorization of ln‐transformed D‐dimer values. This stratification was informed by statistically significant threshold effects identified through segmented regression modeling (Table 3). Specifically, Model II^a^ in the threshold regression analysis revealed an inflection point at K = −1.77 (corresponding to ln D‐dimer levels) beyond which the relationship with tuberculosis dissemination risk demonstrated significant nonlinear characteristics.

Multiple regression analysis (Table 4) revealed a significant independent association between lymphocyte count and D‐dimer levels in tuberculosis patients. In the nonadjusted model, each unit increase in natural log‐transformed lymphocyte count was associated with a substantial 99% lower risk of elevated D‐dimer (OR: 0.01, 95% CI: 0.00–0.04, *p* < 0.001). This inverse relationship remained statistically significant after further comprehensive adjustment for clinical parameters (Adjusted: OR: 0.13, 95% CI: 0.03–0.65, *p* < 0.013), indicating an 87% risk reduction per unit increase in lymphocyte count.

**TABLE 4:** Associations of lymphocyte counts levels and D‐dimer in tuberculosis patient, across different adjustment models.

Exposure	Outcome	Nonadjusted	Adjusted
ln lymphocyte count	ln D‐dimer categorical	0.01 (0.00, 0.04) < 0.001	0.13 (0.03,0.65) < 0.013

*Note:* Results are presented as odds ratios (OR) with 95% confidence intervals (CI) and *p*‐values. Variables were natural logarithmic transformations of D‐dimer and lymphocyte counts level. Adjust model adjusted for Age, AFB smear, T‐SPOT.TB, WBC, HGB, PLT, FBG, ALB, CHOL, ADA, and NK cells.

Abbreviations: ADA, adenosine deaminase; ALB, albumin; CHOL, cholesterol; FBG, fasting blood glucose; HGB, hemoglobin; NK cells, natural killer cells; PLT, platelet; WBC, white blood counts.

Sample sizes:

**Table:** 

Non‐adjusted	Adjust
1900	1900

### 3.6 Curve Fitting Analyses Reveal Distinct Associations Between ln Lymphocyte Counts and ln D‐Dimer

Smooth curve fitting (Figure S3) illustrating the relationship between natural log‐transformed basal lymphocyte counts (*x*‐axis) and natural log‐transformed D‐dimer concentrations (*y*‐axis). The inverse relationship indicates decreasing coagulation biomarker levels with increasing lymphocyte counts across the clinical spectrum. Statistical modeling derived from a cohort of 1900 tuberculosis patients (see Section 2).

### 3.7 Associations of ln Lymphocyte Counts With D‐Dimer Categorization: Linear and Threshold Regression Models

This analysis investigated the relationship between lymphocyte counts (ln‐transformed) and D‐dimer categorization using complementary modeling approaches. Results demonstrate a linear threshold effect in lymphocyte‐associated coagulation activity (Table 5).

**TABLE 5:** Associations of ln lymphocyte counts and ln D‐dimer categorical.

Model	Exposure variable	Outcome variable	Effect value (*β*)	95% Confidence interval (CI)	*p*
Model I	ln lymphocyte counts	ln D‐dimer categorical	0.13	(0.03, 0.65)	< 0.013
Model II	ln lymphocyte counts	ln D‐dimer categorical	−0.25 (Break point)	—	—
	< K		0.00	(0.00, inf.)	0.744
	> K		0.15	(0.03, 0.75)	0.022

*Note:* K: Represents the break point identified in the analysis Inf. refer to Infinity. The *p*‐value less than 0.05 considered significant.

Linear Association (Model I) shows that a significant negative association was observed between lymphocyte count and D‐dimer categorical levels (OR = 0.13, 95% CI: 0.03–0.65; *p* < 0.013). This suggests that each unit increase in lymphocyte count is associated with an 87% reduction in the risk of elevated D‐dimer levels, highlighting its potential protective role.

Model II posits a “threshold (K = −0.25)” for the association, splitting lymphocyte levels into two segments (< K and > K): this threshold (K = −0.25) corresponds to a specific lymphocyte level (≈780 cells/μL). For the < K segment (Effect 1), the effect size is 0.00 (95% CI: 0.00–inf.) with a *p*‐value of 0.7433, which is not statistically significant, indicating no meaningful association between lymphocytes (when below the threshold) and D‐dimer grouping; in contrast, the > K segment (Effect 2) yields an effect size of 0.15 (95% CI: 0.03–0.75) and a *p*‐value of 0.022, which is statistically significant, pointing to a significant association between lymphocytes (when above the threshold) and D‐dimer grouping. The effect difference between these two segments (Effect 2 vs. Effect 1) has a *p*‐value of 0.757 (with an estimate of inf.), confirming that there is no significant difference in effects between the two segments. Additionally, the log‐likelihood ratio test (for model comparison) yields a *p*‐value of 0.469, indicating no significant difference in fit between Model II (the threshold effect model) and Model I (the linear effect model)—meaning the threshold model does not outperform the linear model.

In conclusion. Lymphocyte counts are significantly associated with D‐dimer grouping (supported by Model I and the > K segment of Model II). While Model II identifies a threshold (K = −0.25), this threshold lacks robust statistical support (no segment effect difference, log‐likelihood ratio tests nonsignificant), so a linear association is the more plausible interpretation.

### 3.8 Mediated Effect of D‐Dimer on the Association Between Lymphocyte Count and Risk of PTB + EPTB

We observed a positive association between D‐dimer levels and PTB + EPTB risk, and an inverse association between lymphocyte counts and PTB + EPTB. Furthermore, lymphocyte counts demonstrated a significant negative correlation with D‐dimer levels, suggesting D‐dimer may mediate the relationship between lymphocyte and PTB + EPTB. To formally evaluate this mechanistic pathway, we conducted mediation analysis.

**FIGURE 1:**
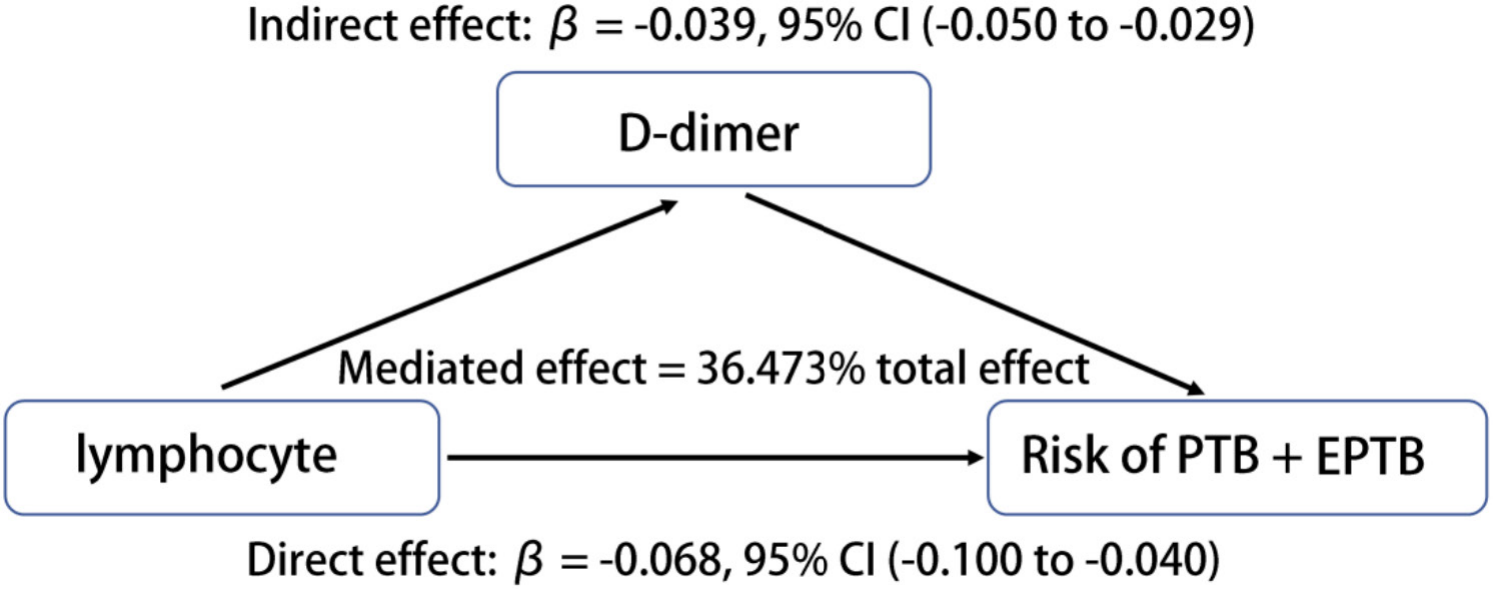
Mediation of D‐dimer on the association between lymphocyte counts and PTB + EPTB risk.

Mediation analysis (Table 6 and Figure 1) demonstrated that a significant direct effect of lymphocyte counts on PTB + EPTB risk (adjusted *β* = −0.068, 95% CI: −0.100 to −0.040; *p* < 0.001), alongside a robust indirect effect mediated by D‐dimer (adjusted *β* = −0.039, 95% CI: −0.050 to −0.029; *p* < 0.001). D‐dimer accounted for 36.473% (95% CI: 25.469–53.168; *p* < 0.001) of the total effect (adjusted *β* = −0.107, 95% CI: −0.140 to −0.078; *p* < 0.001), indicating it serves as a key mechanistic pathway linking lower lymphocyte counts to elevated PTB + EPTB risk, after adjustment for AFB smear, age, hematological, and metabolic confounders.

**TABLE 6:** Mediation analysis of the association between lymphocyte counts and risk of PTB + EPTB mediated by D‐dimer.

Model	Nonadjusted		Adjusted	
Effect type	*β* (95% CI)	*p*	*β* (95% CI)	*p*
Direct effect	−0.051 (−0.078, −0.024)	< 0.001	−0.068 (−0.100, −0.040)	< 0.001
Indirect effect	−0.086 (−0.101, −0.072)	< 0.001	−0.039 (−0.050, −0.029)	< 0.001
Total effect	−0.137 (−0.165, −0.111)	< 0.001	−0.107 (−0.140, −0.078)	< 0.001
PM (%)	62.752 (50.511,78.876)	< 0.001	36.473(25.469,53.168)	< 0.001

*Note:* PM (proportion mediated): D‐dimer accounted for 36.473% of the total effect of lymphocyte counts on risk of PTB + EPTB. Adjusted for: Age, AFB Smear, WBC count, HGB, PLT, FBG, ALB, CHOL, T‐SPOT.TB; ADA; NK.

Abbreviations: ADA, adenosine deaminase; ALB, albumin; CHOL, cholesterol; FBG, fasting blood glucose, HGB, hemoglobin; NK cells, natural killer cells; PLT, platelet; WBC, white blood counts.

## 4 Discussion

In this large retrospective cohort study of 1900 tuberculosis patients (2022–2024), we investigated lymphocyte counts as a protective exposure variable, D‐dimer as a mediating biomarker, and incident combined pulmonary–extrapulmonary tuberculosis (PTB + EPTB) as the outcome. Leveraging causal mediation analysis—a methodological advance beyond correlative approaches—our findings quantitatively demonstrated that D‐dimer mediated a significant 36.473% of the protective effect of high lymphocyte counts against PTB + EPTB risk, providing actionable thresholds (D‐dimer > 0.170 mg/L and lymphocytes < 750 cells/μL) for clinical intervention.

Our findings establish lymphopenia as an independent risk factor for PTB + EPTB, corroborating earlier retrospective observational studies [24]. Notably, among hospitalized EPTB patients, HIV coinfection and low CD4◻ T‐cell counts were significantly associated with meningeal and disseminated disease, though this observation was limited to 320 cases [24]. Further supporting our results, Tang et al. [11] reported diminished CD4◻ T‐cell proportions and reduced CD4◻/CD8◻ ratios in 587 HIV‐negative tuberculosis patients with disseminated infection. While their findings align with ours, our study benefits from a substantially larger cohort. Similarly, Sun et al. [25] observed markedly lower lymphocyte counts—including CD4◻ T cells, CD8◻ T cells, and NK cells—and impaired lymphocyte function in active TB patients compared to those with latent tuberculosis infection (LTBI) or healthy controls. However, their case–control study (*n* = 171) contrasted active TB with LTBI and healthy individuals, whereas our work specifically stratifies disease severity among active TB patients, comparing isolated PTB with the more severe PTB + EPTB presentation. This approach provides novel insights into lymphocyte depletion as a biomarker for disseminated disease progression.

Our investigation also reinforces the prognostic role of D‐dimer in TB. Li et al. [26] documented elevated D‐dimer levels (≥ 0.5 mg/L) in 75% of tuberculous pleural effusion patients. While prior studies have explored D‐dimer in various TB contexts, ours is the first to focus on its role in PTB with EPTB involvement. Earlier research includes Liu et al.'s use of D‐dimer and CRP to differentiate bacterial from tuberculous meningitis [27], and Suryakusumah et al.'s demonstration of elevated D‐dimer levels in advanced TB lesions [28] Consistent with our findings, lymphopenia was strongly associated with increased D‐dimer levels, indicative of poor prognosis.

This lymphocyte‐D‐dimer relationship extends beyond TB, as evidenced by Shen et al.'s meta‐analysis [29], which linked higher D‐dimer and lower lymphocyte subsets to severe COVID‐19 infection. A similar trend was observed in a retrospective analysis of 187 colorectal cancer patients, further underscoring the clinical relevance of these biomarkers.

Mechanistically, The lymphopenia observed in disseminated tuberculosis is attributed to the sequestration and exhaustion of peripheral blood lymphocytes in infected tissues, such as the lung parenchyma or extrapulmonary sites of severe disease involvement [30]. This reduction, particularly in CD4◻and CD8◻ T‐cell subsets, compromises granuloma integrity, facilitating mycobacterial dissemination to extrapulmonary sites [31]. While the precise link between lymphopenia and coagulopathy remains incompletely understood, the negative correlation between lymphocyte counts and D‐dimer levels is supported by experimental evidence involving plasminogen activator inhibitor Type‐1 (PAI‐1). PAI‐1 deficiency in murine models increases fibrin degradation products (D‐dimer) [32] while impairing lymphocyte cytokine responses (IFN‐*γ*, TNF‐*α*, IL‐4, IL‐6, and IL‐10) upon tuberculosis infection [33]. This dual effect—elevated D‐dimer with suppressed lymphocyte function—provides a mechanistic basis for our clinical findings. Furthermore, clinical studies confirm that high D‐dimer levels correlate with reduced lymphocyte blast transformation in disseminated tuberculosis patients [34]. Parallels can be drawn to severe COVID‐19, where dysregulated immune responses—driven by cytokine storms (e.g., TNF‐*α* and IL‐6), lymphocyte apoptosis, hypoxia, and endothelial damage—contribute to coagulation activation [35]. In disseminated TB, the loss of lymphocyte‐mediated control allows mycobacterial spread, which in turn induces a proinflammatory cytokine storm. These cytokines upregulate tissue factor expression, activating coagulation cascades and elevating D‐dimer levels [24]. Notably, D‐dimer may not only serve as a marker of hypercoagulability but also exacerbate inflammation and micro thrombosis, further promoting bacterial dissemination. Thus, lymphopenia → coagulopathy → D‐dimer elevation → dissemination forms a plausible pathogenic axis in advanced TB.

This study establishes lymphocyte count (< 750 cells/μL) and D‐dimer (> 0.170 mg/L) as critical thresholds for predicting PTB progression to EPTB, reaffirming the role of lymphopenia in dissemination while pioneering the identification of D‐dimer as an independent risk marker. Beyond mere association, we demonstrate—for the first time—that 36.473% of lymphocytes' protective effect is mediated by D‐dimer, mechanistically linking immune dysfunction (lymphocyte depletion) to coagulopathy (D‐dimer elevation) and ultimately to bacterial dissemination.

By employing causal mediation analysis in a large, rigorously adjusted cohort, we bridge a longstanding gap in TB pathophysiology, proposing the lymphocyte → D‐dimer → dissemination axis as a unifying framework. Clinically, these thresholds offer a practical tool for risk stratification: patients crossing either threshold warrant intensified monitoring (e.g., imaging, fibrinolytic markers) to preempt extrapulmonary spread. Future studies must validate these cutoffs globally and dissect immune‐fibrinolytic cross talk to guide targeted interventions—whether immunostimulatory (e.g., IL‐2 to boost lymphocytes) or anticoagulant (e.g., heparin to curb D‐dimer effects).

This study has limitations, including single‐center design and potential selection bias. Additionally, data loss occurred due to certain diagnostic tests not being covered by medical insurance, which may have prevented comprehensive initial patient assessments. While we have provided mechanistic evidence from existing literature [32, 33, 34] to support the biological plausibility of the lymphocyte‐D‐dimer association, direct experimental validation of this specific pathway in tuberculosis was beyond the scope of this clinical study. Future experimental studies, particularly those utilizing tuberculosis‐specific animal models, are needed to confirm the causal relationships suggested by our clinical findings. Multicenter studies are needed for validation.

## 5 Conclusion

This study demonstrates that D‐dimer is an independent risk factor while lymphocyte count is a protective factor against tuberculosis dissemination. Crucially, causal mediation analysis revealed that D‐dimer mediates 36.473% of the protective effect of lymphocytes. The identified actionable thresholds (D‐dimer > 0.170 mg/L and lymphocytes < 750 cells/μL) provide a critical tool for clinical risk stratification, enabling early intervention to prevent extrapulmonary dissemination in patients with pulmonary tuberculosis. This finding provides a novel mechanistic link, proposing a pathogenic axis where lymphocyte depletion leads to coagulopathy (evidenced by elevated D‐dimer), which in turn facilitates bacterial spread.

## Author Contributions

**Yujun Lin:** funding acquisition, data curation, formal analysis, investigation, resources, software, visualization, and writing – original draft. **Di Wu:** validation. **Xiaohong Chen:** funding acquisition, conceptualization, methodology, project administration. **Lujing Jiang:** writing–review and editing. **Yiming Zeng:** conceptualization, methodology, project administration, supervision, and writing – review and editing. All authors have read and agreed to the published version of the manuscript.

## Funding

The work was supported by the Clinical Key Specialty Construction Project Funding of Fuzhou City, the Provincial Clinical Key Specialty Construction Project of Fujian Province (No. 20230104), and the Medical Technology Innovation Project (No. 2024‐YC‐023) under the Social Development category of Municipal Science of Fuzhou City, Fujian Province, China.

## Ethics Statement

This retrospective study was approved by the Institutional Review Board of Fuzhou Tuberculosis Prevention and Control Institute, Fujian Province (approval number: 2023‐010 (Research)‐02).

## Consent

Informed consent had been obtained from all patients at the time of hospital admission, allowing the use of their deidentified clinical information for clinical research purposes.

## Conflicts of Interest

The authors declare no conflicts of interest.

## Supporting information


**Figure S1:** Patient recruitment flowchart.
**Figure S2:** Nonlinear associations of biomarkers with PTB + EPTB risk.
**Figure S3:** Nonlinear associations between ln lymphocyte counts and ln D‐dimer.

## Data Availability

The data that support the findings of this study were obtained from Fuzhou Pulmonary Hospital of Fujian Province, China. The data are available from the corresponding author upon reasonable request.
