# Supplementary material for: D‐Dimer: A Mediator of the Association Between Lymphocyte and Dissemination of Pulmonary Tuberculosis: A Retrospective Cohort Study
Source: Clin Respir J. 2026 Mar 17;20(3):e70175. doi: 10.1111/crj.70175 (PMC13093727; doi:10.1111/crj.70175)
Supplement: Supplementary file 1 — Figure S1: Patient recruitment flowchart. Figure S2: Nonlinear associations of biomarkers with PTB + EPTB risk. Figure S3: Nonlinear associations between ln lymphocyte counts and ln D‐dimer. [file CRJ-20-e70175-s001.docx]

**Supplementary Figure 1** Patient Recruitment Flowchart


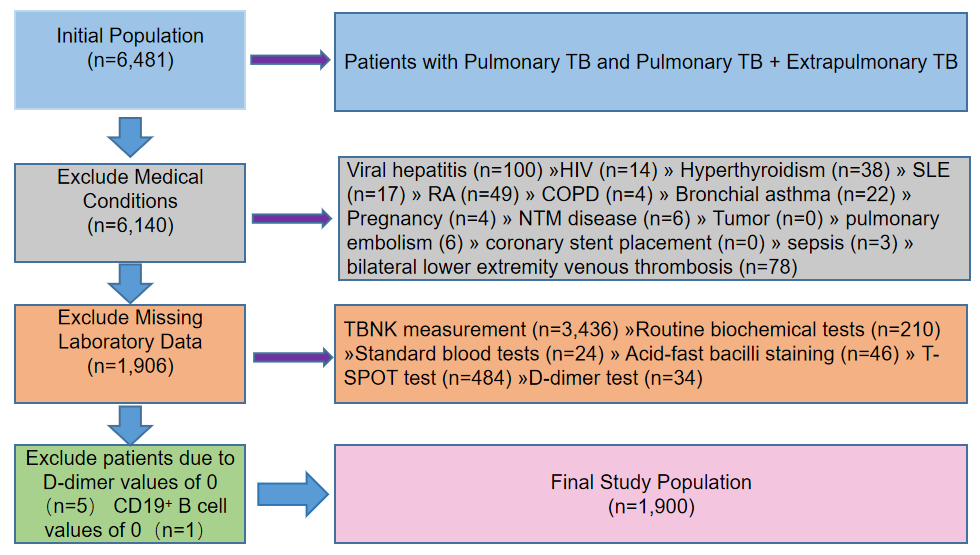


» indicates the next step is to exclude; Chronic Obstructive Pulmonary Disease (COPD); Nontuberculous Mycobacteria (NTM); TBNK analysis included CD3^+^ , CD4^+^ , CD8^+^ T cells, CD19^+^B cells and CD16^+^CD56^+^ NK cells counts; Routine biochemical tests included fasting blood glucose (FBG), albumin (ALB), cholesterol (CHOL), and adenosine deaminase (ADA); Standard blood tests included white blood cell (WBC), lymphocyte (LYM), platelet (PLT) counts and hemoglobin (HGB).

**Supplementary Figure 2** Nonlinear Associations of Biomarkers with PTB+EPTB Risk.


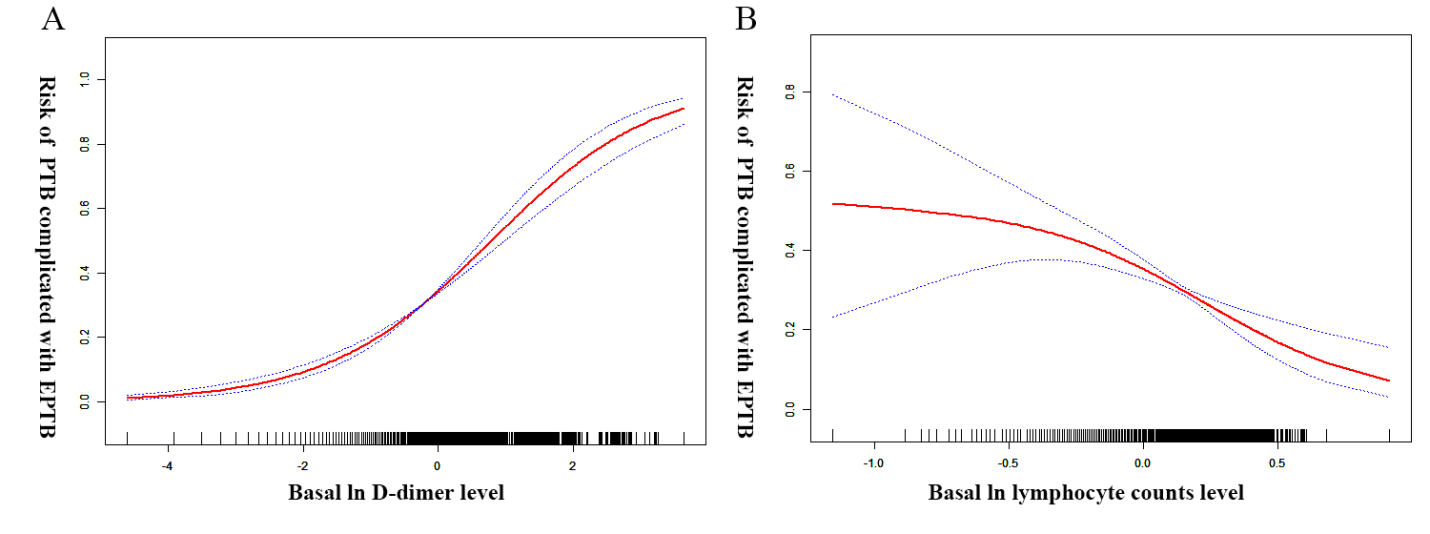


The red solid line represents the predicted relationship from generalized additive models, with blue dashed lines indicating 95% confidence intervals. Tick marks at the base show ln D-dimer and ln lymphocyte count distribution density.

**Supplementary Figure 3** Nonlinear Associations between ln lymphocyte counts and ln D-Dimer.





The red solid line represents the predicted relationship from generalized additive models, with blue dashed lines indicating 95% confidence intervals. Tick marks at the base show ln lymphocyte count distribution density.
